# Scheduling Group Care in Routine Perinatal Care: Identifying Implementation Modifications Across Belgium, Kosovo, and the UK

**DOI:** 10.3390/healthcare14121642

**Published:** 2026-06-10

**Authors:** Astrid Van Damme, Florence Talrich, Deborah L. Billings, Christine McCourt, Ashley Gresh, Crystal L. Patil, Matty Crone, Marlies Rijnders, Ilir Hoxha, Sharon Schindler Rising, Katrien Beeckman

**Affiliations:** 1 Nursing and Midwifery Research Group (NUMID), Faculty of Medicine and Pharmacy, Primary Care (PRIM), Vrije Universiteit Brussel (VUB), Universitair Ziekenhuis Brussel (UZ Brussel), Laarbeeklaan 101-103, 1090 Brussels, Belgium; 2 Department of Health Promotion, Education, and Behavior, Institute for Families in Society, University of South Carolina, Columbia, SC 29208, USA; 3 Group Care Global, Philadelphia, PA 19119-3739, USA; 4 Centre for Maternal and Child Health Research, School of Health and Medical Sciences, City St George’s, University of London, London EC1V 0HB, UK; 5 Johns Hopkins School of Nursing, Baltimore, MD 21205, USA; 6 School of Nursing, University of Michigan, Ann Arbor, MI 48109, USA; 7 Department of Health Promotion, Maastricht University, 6229 HA Maastricht, The Netherlands; 8 TNO Child Health, 2333 BE Leiden, The Netherlands; 9 Evidence Synthesis Group, Prishtina Stars, 10000 Prishtina, Kosovo

**Keywords:** modifications, group care, implementation, antenatal care, FRAME

## Abstract

**Background**: Group Care (GC) is an antenatal/postnatal care model comprised of a stable group of pregnant people or parent–child dyads receiving care in two-hour group sessions that combine clinical care with interactive discussion and learning. Integrating GC into healthcare systems organised for individual care poses challenges at both site and system levels. This study identified scheduling-related modifications across contexts to understand modification processes. **Methods**: We used an explanatory sequential design with mixed qualitative methods across seven GC implementation sites in Belgium, Kosovo, and the United Kingdom. A qualitative survey based on the Framework for Reporting Adaptations and Modifications to Evidence-based interventions (FRAME) was completed at each site by multiple stakeholders. Subsequently, in-depth interviews were conducted to further explore modification processes and examine whether changes were sustained or discontinued up to three years post-implementation initiation. **Results**: Two modifications were identified across countries: (1) combining GC sessions with individual consultations, and (2) integrating GC into digital booking and medical record systems. Guided by FRAME, we identified similarities and differences in the goals, drivers, and impact of these modifications. The dominant one-to-one antenatal care model strongly influenced modifications, making it more difficult to implement GC as a stand-alone model in obstetrician-led systems (Belgium and Kosovo) compared to a midwifery-led system (UK). In both contexts, the dominant model negatively influenced the perceived value of GC, with GC sessions viewed only as education and individual consultations seen as the actual care. Integration in the booking system appeared essential for payment and scheduling arrangements. **Conclusions**: Integrating GC scheduling into existing care pathways is challenging in systems where one-to-one care is the predominant model. Sustainable integration of GC requires early coordination and shared ownership across areas, including clinical, administrative, and IT.

## 1. Introduction

Complex health interventions have multiple interacting components, target a range of outcomes, and require flexibility in their implementation [1,2]. Implementing these interventions within existing healthcare systems, because of their well-established bureaucratic processes, is widely recognised as challenging [3,4,5]. ‘Scheduling’ is a critical component of implementation. This broad concept operates at different layers of the healthcare system. At the site level, scheduling involves the logistical coordination of scheduling patients’ appointments and the organisation of the providers’ work schedules and spaces [6,7]. At a more macro level, scheduling refers to fitting the intervention within already established mechanisms of care, especially existing local and national care pathways and guidelines [7,8,9]. Therefore, scheduling complex health interventions, both within implementation sites and the broader health care system, is not merely a logistical issue but a core implementation challenge that affect the intervention’s feasibility, fidelity, and sustainability.

Because complex health interventions are highly context-sensitive, they are rarely implemented without changes to their original design or delivery. However, less is known about how specific changes occur during implementation, particularly within core operational processes such as scheduling. These changes may take the form of adaptations, defined as purposeful and planned alterations intended to improve the contextual fit of an intervention while preserving its core components [10]. They can also be modifications, which represent a broader category that also includes unintentional and/or reactive changes that occur during the process of real-world implementation [11]. Throughout this study, the term ‘modifications’ is used because this encompasses both adaptations and reactive unplanned changes.

Antenatal and postnatal Group Care (GC), based on the CenteringPregnancy^®^ and CenteringParenting^®^ model, is an example of a complex health intervention that is increasingly implemented across diverse healthcare systems worldwide. GC combines three core components—health assessment, interactive learning, and community building—and is enacted through group-based maternal and child healthcare [12]. Research has demonstrated high satisfaction among both care users and providers [13,14,15] as well as positive effects on several maternal and child health outcomes [16,17,18,19,20,21,22,23,24]. It has also shown positive effects on health literacy [25]. The World Health Organization (WHO) has identified GC as a promising strategy to enhance the quality and equity of maternal and child healthcare [26].

Despite its promising results, GC is associated with substantial implementation challenges [27,28,29,30,31]. GC requires a reorganisation of routine care delivery, as it replaces or is offered as an alternative to one-to-one antenatal or postnatal consultations [32,33,34]. Previous contextual analyses conducted within the “Group Care for the first 1000 days” implementation research project (GC_1000) [35] identified scheduling GC within routine care pathways as an anticipated challenge to its implementation [36].

In this context, scheduling refers to the alignment of GC sessions with existing care trajectories at both the local level (i.e., site-specific organisation, alignment with local guidelines and facilitators’ workflows) and the national level (i.e., alignment with protocols and national guidelines). Because perinatal care pathways are often shaped by local practices, national regulations, and political decisions, scheduling is not merely a logistical task but a complex element of implementation. These findings are consistent with the broader global literature, which repeatedly reports that embedding GC within existing healthcare structures is challenging and that those challenges may influence successful adoption and continuation [27,37,38]. Moreover, previous research within the GC_1000 project demonstrated that implementation involves close collaboration among multiple professionals, such as midwives, physicians, psychologists, social workers, health visitors, administrative staff, and managers [36,39]. In line with the GC model, this collaboration is often structured through a local steering committee, typically composed of a mix of clinical, administrative, and other relevant stakeholders, including patients, who support and guide the implementation process at the site level [12]. Scheduling is particularly relevant because it represents the intersection of organisational structures, professional roles and boundaries, possibilities for multidisciplinary collaboration, and patient access to care. Together, these considerations suggest that scheduling is a key domain where modifications occur during implementation since the intervention must be adjusted to align with local workflow and system structures.

### Aim and Research Focus

The aim of this study is to generate insight into how scheduling for a complex health intervention, namely GC, was offered as an alternative to individual routine care in implementation sites where antenatal care is delivered in Belgium, Kosovo, and the UK. More specifically, this study seeks to examine:

1. what modifications related to the scheduling of GC occurred across three countries
2. the processes through which the modifications were made

## 2. Methods

### 2.1. Design

An explanatory sequential design with mixed qualitative methods was used for this study. The study was part of the broader GC_1000 research project, in which 24 implementation sites across seven countries implemented antenatal and/or postnatal GC. For this part of the study, we focus on the participating sites from Belgium (N = 3), Kosovo (N = 2), and the United Kingdom (UK) (N = 2), which include six urban sites implementing antenatal GC, and one site in the UK that implemented continuous antenatal and postnatal GC. Table 1 presents the participating countries’ characteristics.

Within this study, GC was implemented in two primary care sites and one hospital in Belgium. In Kosovo, it was implemented in two hospitals. In the UK, the implementation took place in one hospital, and one cooperation between antenatal care service with a health visitor postnatal care service. Routine antenatal care in the UK is midwifery-led. In both Belgium and Kosovo, routine antenatal care is obstetrician-led, although midwives are legally allowed to conduct pregnancy follow-up. The GC implementation plan in Belgium consisted of six/seven antenatal GC sessions facilitated by midwives, complemented by three individual consultations with an obstetrician. In Kosovo, the model included eight antenatal GC sessions facilitated by midwives, supplemented with monthly individual consultations with an obstetrician. In the UK, the GC model comprised 9 antenatal GC sessions organised by midwives; participants were referred to an obstetrician only in cases of pregnancy complications or risks, in accordance with standard care protocols. In all countries, the total number of antenatal care contacts (either GC sessions and/or obstetrician contacts) were in line with national antenatal care guidelines. More detailed information on the participating sites is described in the studies from Martens et al. [35], Van Damme et al. [36], and in McCourt et al. [40].

### 2.2. Data Collection

**Figure 1:**
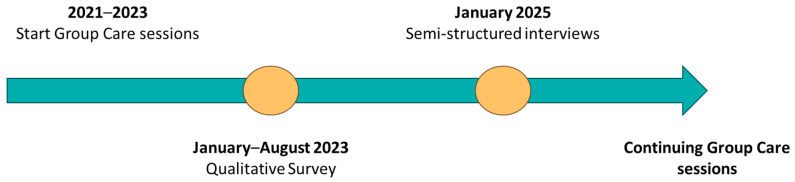
Timeline of data collection.

Data were collected at two time points: a qualitative survey was conducted approximately one year after the initiation of the GC sessions, followed by in-depth interviews between two and three years after the start of the sessions (Figure 1) [41].

First, a qualitative survey incorporating open- and closed-ended structured questions was conducted. We structured the qualitative survey using the Extended Framework for Adaptations and Modifications to Evidence-based Interventions (FRAME) constructs and predefined response options for capturing adaptations and modifications to GC scheduling (see Supplementary File S1) [42]. The FRAME offers a structured approach for capturing multiple aspects of the modification process, addressing not only the modifications themselves but also the underlying mechanisms and context that shape them. More specifically, it comprises a series of core constructs that facilitate the systematic description and interpretation of intervention modifications. These constructs address multiple dimensions of modifications, including what component was modified, its timing, whether changes were planned or reactive, and who was involved in the decision-making process. In addition, the FRAME captures the nature of the modification, the level at which it was modified (i.e., for whom or for what the modification was made), the contextual domain affected (such as format, setting, personnel, or target population), its relationship to intervention fidelity (i.e., are core functions preserved or changed), and the underlying goals and reasons for the modification. The survey is available in Supplementary File S2. Each construct of the FRAME is assessed using closed multiple-choice questions, followed by open-ended questions to capture additional contextual information and to allow participants to elaborate on their responses from their own perspective. This approach leverages the strengths of a qualitative survey by combining a clearly defined research focus with the flexibility for participants to provide detailed, nuanced, and contextual insights [41]. The survey was conducted through Qualtrics XM version 2.78.1 (Qualtrics, Provo, UT, USA), a programme for online surveys.

To build on the qualitative survey data and gain an in-depth understanding of the results, semi-structured interviews were conducted following a preliminary analysis of the survey responses. Conducting the preliminary analysis prior to conducting the interviews enabled the research team to identify remaining gaps in the data and areas requiring further clarification to support a thorough and accurate interpretation of the findings. Prior to each interview, a tailored topic guide was developed, with questions specifically adapted to the individual interviewee and their site context. These targeted questions were designed to elicit additional detail and clarification on issues that were not fully captured in the survey data, thereby ensuring that the interviews directly contributed to answering the research questions. Furthermore, the interviews provided insights into whether the modifications described in the survey were continued. All interviews were conducted online by the corresponding author, a PhD candidate from Belgium who was involved in the GC_1000 research from its inception in 2020, and who conducted over 80 interviews with participants in seven countries. In addition, the interviewer, a midwife researcher, was trained in GC and has published on GC implementation. To enhance reflexivity and reduce the risk of single-perspective researcher bias, the study was supported by co-authors from multiple countries, including those participating in the research. These researchers have extensive expertise in collecting and analysing qualitative data and in-depth knowledge of the GC model. All were involved in developing the interview topic guides, data interpretation and analysis. The interviewer had previously interviewed all interviewees in a previous stage of the research to conduct a context analysis of the GC implementation context. While this prior relationship may have introduced a risk of socially desirable answers, it was also considered beneficial for building on established trust and facilitating open discussion about complex implementation processes. To minimise potential bias, there were no collegial or hierarchical relationships between the interviewer and participants, interviewees remained anonymous in the reporting, and questions were formulated to explicitly invite critical reflection and discussion of challenges. Participants were informed about the interviewer’s background and the focus of the interview, and were reminded that participation was voluntary, that interviews could be stopped at any time, and that withdrawal from the study remained possible after the interview without consequence. All interviews were recorded after obtaining oral consent from the interviewees, in accordance with ethical guidelines.

### 2.3. Sampling

Between January and August 2023, the online qualitative survey was administered to three respondents at each of seven implementation sites across the three participating countries. A purposive sampling strategy was employed to ensure representation of three predefined respondent profiles within each site: (1) one GC facilitator serving as a site-level implementer; (2) one member of the national GC_1000 research team, representing project-level implementers with close links to the site; and (3) one Group Care Global (GCG) consultant involved in supporting GC implementation and training at the site. All selected participants completed GC training and were actively engaged in the implementation and/or evaluation of GC as part of the GC_1000 project. This respondent selection aligns with the principles of qualitative survey research, which prioritizes the inclusion of diverse relevant perspectives to enhance depth of understanding rather than achieving statistical representativeness or generalisability [41,43]. By incorporating different perspectives within each site, and across sites in different countries, we aimed to develop a more nuanced and comprehensive understanding of the modifications.

For each site, the interviewees were identified in consultation with the GC_1000 researchers of the included countries to select the most suitable respondents to address the research questions. Rather than aiming for high numbers of interviewees or data saturation, sampling was guided by the concept of information power [43]. Key factors of this concept (including study aim; study specificity; use of theory; quality of dialogue; and analysis strategy) contributed to the estimation of the sample size: the study aim is narrow and focused on scheduling GC within routine services; the sample is highly specific, including participants with unique, context-specific expertise in GC implementation; and the study is theoretically grounded in the FRAME, which clearly defines the domains of interest. A high-quality dialogue was anticipated given the interviewer’s experience and the specific knowledge of the interviewees. Together, these elements ensure sufficient information power to address the research questions including a small sample size [43].

### 2.4. Data Analysis

As a first step in the analysis, all survey data were transferred into SPSS software, version 29.0.1.0 (IBM Corporation, Armonk, NY, USA). A deductive preliminary data-analysis was conducted, in which responses were organised according to the FRAME core constructs and dimensions. Following the identification of the modifications occurring in multiple countries, and a preliminary analysis of these data, we identified gaps in the survey data to better understand the scheduling modification processes and to develop the interview topic guides. Following the interviews, an inductive thematic analysis was conducted on the survey data, along with the verbatim interview transcripts, focusing on the modifications identified [44]. After integrating all data of the FRAME elements and additional data from the interviews, we thematically organised our insights in the modification process into three overarching components: (1) the underlying goal or purpose that motivated implementers to introduce this modification, (2) the key drivers underpinning the introduction of this modification, and (3) the positive/negative/unintended impact that these modifications had on GC implementation from the respondent’s perspective. In line with the recommendations by Braun et al. [41], the analysis moved beyond a purely deductive, question-by-question summarisation of qualitative survey responses and instead approached the dataset holistically.

## 3. Results

### 3.1. Responses

A total of 19 surveys were completed, and five follow-up interviews conducted (Table 2). Nearly all invited respondents completed the survey. One UK facilitator response was missing due to staff shortages. In Kosovo, one GCG consultant completed the questionnaire jointly for both sites by mutual agreement. In Belgium, two of the interviewees were site coordinators of the GC_1000 project, while in the third site a facilitator directly involved in implementation was interviewed. In Kosovo, the interview participant was a researcher who was connected to the implementation sites and conducted multiple observations of GC sessions within the GC_1000 project. In the UK, the interviewee was also a researcher who was involved in the GC_1000 project, maintained close contact with implementation sites, and served as a GC trainer.

### 3.2. Cross-Country Modifications Regarding Scheduling

Across the three countries, respondents described a range of the most impactful modifications regarding scheduling during GC implementation. In total, two scheduling-related modifications were identified through the qualitative survey that are reported in at least two of the three countries: (1) the combination of GC sessions with individual consultations within the perinatal care pathway, and (2) the integration of GC into the IT booking system for antenatal consultations.

### 3.3. Combination of Group Care and Individual Consultations: The Best of Two Worlds?

**Figure 2:**
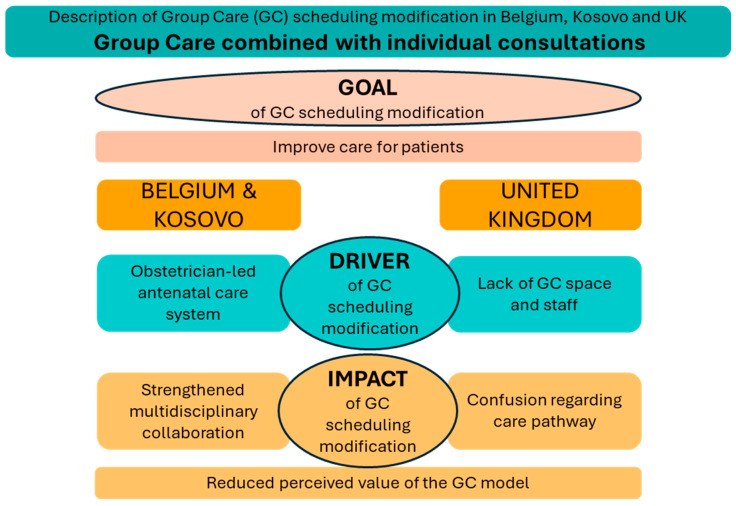
Goal, drivers, and impact of the combination of Group Care with individual consultations in Belgium, Kosovo and United Kingdom.

Across all three participating countries, a combination of GC sessions with individual appointments as antenatal care trajectory was described as the most impactful scheduling-related modification of the planned GC implementation (see Table 1). We identified similarities and differences between the countries related to the goal, driving factors and impact of this modification, as presented in Figure 2.

#### 3.3.1. Description of the Modification

In Belgium, from the outset of implementation, it was anticipated that GC would be combined with three individual consultations with obstetricians, given the central role of obstetricians in antenatal care and the need for ultrasound examinations, which midwives are not authorised to perform. However, in practice, GC resulted in a duplication of care: while the planned number of GC sessions was implemented, the number of obstetric consultations was often not reduced. As a result, GC was often offered on top of routine care rather than as a replacement, as described in the survey:

*“They had planned changes in the number of Group Care sessions (fewer sessions) and planned fewer consultations with the obstetrician. But, in practice, the obstetricians didn’t reduce their individual consultations. Therefore, Group Care is more additional care.”*

(Survey, Belgium)

Similar to Belgium, GC was implemented as an addition to routine obstetrician-led care in Kosovo. In response to attendance challenges, GC facilitators adapted the schedule by organising GC sessions on the same day as individual obstetric consultations:

*“In the beginning it was not planned. It came up later, after women started to complain that it is not possible for them to come in two different days for receiving medical services. This is why we decided to help them [the participants] in this form”*

(Survey, Kosovo)

As in Belgium, this led to a situation where GC in the sites in Kosovo did not replace but rather complemented existing care.

In contrast, in the UK, where antenatal care is predominantly midwifery-led, the modification took a different form. In one participating site, three GC sessions were replaced by individual consultations with the midwife who facilitates the GC sessions, thereby guaranteeing some level of care continuity. This resulted in a reduced number of GC sessions overall, with individual consultations embedded within the care pathway.

#### 3.3.2. Goal

All three countries started with the same goal for this modification: to improve care for their patients. In the UK, combining GC with individual consultations was framed as a potential way to turn logistical constraints into an advantage. As an interviewee mentioned: maybe it could be the best of two worlds? In Belgium and Kosovo, it was a pragmatic approach given the strong position of obstetricians in those countries.

#### 3.3.3. Drivers

The underlying drivers for this modification differed among the countries. In the UK, this was mainly driven by logistical constraints, especially limited physical space to organise group sessions, as well as staff shortages. In contrast, in Belgium and Kosovo, the modification was primarily driven by the dominant medicalised model of antenatal care, including the strong position and status of obstetricians. Our interviews highlighted the importance of obstetricians’ support for recruitment and for building trust in both the GC model and the midwife-led sessions. In Kosovo, one site reported strong support and active referrals from obstetricians, whereas collaboration was more limited in another site, as illustrated by the following quote:

*“But in other sites they [obstetricians] didn’t accept it very well. What I heard from the midwives, the facilitators of the group care program, is that they see it like they [the GC facilitators] are interrupting their work [from the obstetricians], like they are interfering in their field. […] And some of them [obstetricians] supported the Group Care program because they knew what they [the GC facilitators] are doing there, the aim of the Group Care.”*

(Interview, Kosovo)

Similarly, in Belgium, limited initial support from obstetricians was reported in two of the three sites, with midwives needing to advocate strongly for a place within the routine antenatal care pathway, as reflected in the following quote:

*“The care pathway for women taking Group Care is being partially integrated into a long-standing negotiation of the prenatal care pathway and remains one of the most challenging aspects of group care in my opinion.”*

(Survey, Belgium)

#### 3.3.4. Impact

Next to different drivers, we also identified differences in the impact of the modifications from the respondents’ perspectives. First, in Belgium and Kosovo, combining GC with individual consultations appeared to foster multidisciplinary collaboration over time. In one Belgian site, different disciplines involved in perinatal care were engaged in decisions about how the GC pathway should be integrated into existing antenatal care structures from the start. This way, they could develop a GC schedule that would fit all their schedules and preferences. An interviewee from another Belgian site clarified that, over time, they did not prioritise reducing obstetric consultations, and relationships between obstetricians and midwives delivering GC improved substantially. Meetings are now systematically scheduled in which the GC facilitators and obstetricians discuss the care of the GC participants, express mutual appreciation, and identify opportunities for complementarity in care provision, as stated by the following participant:

*“It was nice to hear, what the obstetrician said the previous time: ‘Yes, I’m so happy that you are working on that, on the psychosocial issues, because I was also worried about that.’ And I thought, yes, okay, so they also feel that we [GC facilitators] are an added value for them [the obstetricians], which we certainly also feel about them.”*

(Interview, Belgium)

In another Belgian site, GC implementation relies strongly on multidisciplinary collaboration, with general practitioners referring pregnant women and a community health centre providing space for sessions. Over time, this collaboration also offered emotional support during a challenging implementation trajectory, as illustrated by the following interviewee:

*“What is also important there, is the emotional support. Because for the midwives… it does require quite something to prepare in the Group Care way. Especially since we work with vulnerable groups, you know. These are sometimes quite complex situations that demand a lot from the midwife, both in terms of time, effort, and emotionally. … And then we hear from the physicians, ‘Yes, but you’re doing well, you’re doing really well.’ These are physicians we can call or with whom we can vent, like, ‘gosh, this is a difficult case’, you know?”*

(Interview, Belgium)

At the same time, respondents across all countries reported that reducing the number of GC sessions limited the amount of content that could be covered and affected opportunities for group bonding. Furthermore, combining GC with individual care was associated with a negative influence on the perceived value of the GC model, as it was no longer experienced as a comprehensive alternative to individual care. This is illustrated by the following quote from a UK interviewee:

*“So, and it ended up meaning that the midwives ended up doing a lot of additional one-to-one appointments because people started thinking of the groups as education and thinking of the appointments as care.”*

(Interview, UK)

In the UK, a survey respondent mentioned that women were repeatedly missing the last GC session when an individual appointment was scheduled shortly after. The interviewee expressed that combining care modalities also led to confusion among participants regarding the perinatal care pathway:

*“Having some one-to-one and some groups did not turn out to be the best of all worlds. What it turned out to do is firstly: it did affect the bonding of some of the groups because they didn’t get to know each other as well. But I think more importantly, it created a lot of confusion.”*

(Interview, UK)

In Kosovo and Belgium, respondents indicated that without this combination of care, there would be little room within their obstetrician-led healthcare systems to organise GC. The following quote illustrates the influence of these cultural and policy-related factors:

*“It is the cultural norm to have medical follow-up of the pregnancy by an obstetrician; they can’t be left out of the schedule. Midwives are not allowed to conduct ultrasounds in Belgium.”*

(Survey, Belgium)

The interviews provided extra information on the (dis)continuation of the applied modifications. In the UK, where the initial rationale for combining GC with individual sessions was primarily logistical within an otherwise supportive midwifery-led system. This modification was later reversed, and the GC model transitioned back to a fully GC-based care pathway. In Kosovo, the antenatal GC was stopped due to recruitment issues. In Belgium, in two of the three sites, antenatal GC was still running at the time of the interviews, often in addition to individual care offered by the obstetrician.

### 3.4. Digital Integration of GC in the Booking and Medical Record System

#### 3.4.1. Description of the Modification

**Figure 3:**
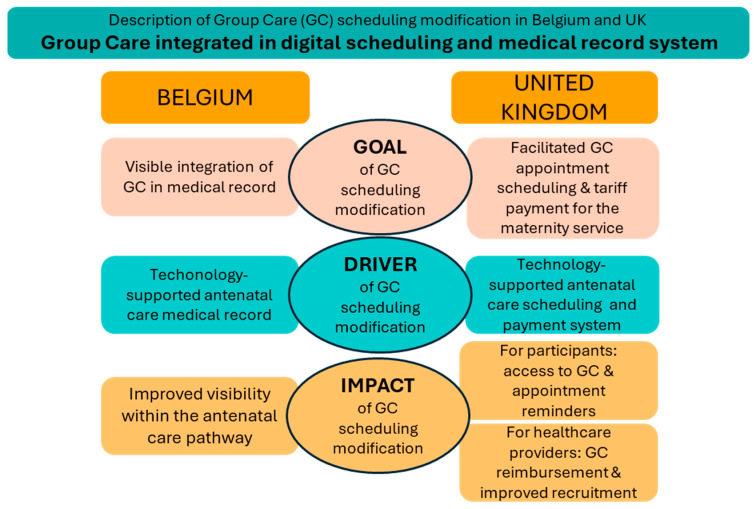
Goals, drivers and impact of the integration of Group Care in the digital scheduling and medical record system in Belgium and United Kingdom.

Both in one site in Belgium and in the UK, integrating GC sessions into the digital scheduling system and the electronic medical record was described as an impactful modification regarding scheduling GC into routine care. In Kosovo, this modification was not mentioned. At one Belgian site, a separate care pathway for GC was established and made visible in the participants’ medical records. This is also visible to the hospital’s obstetricians during individual consultations and at the time of delivery, ensuring that they are informed about the GC trajectory of their patients. In the UK, the GC pathway is fully embedded within the routine scheduling system. Figure 3 illustrates the goals, drivers, and impact identified in relation to this modification.

#### 3.4.2. Goal

In UK, the goal was twofold: to facilitate GC bookings and to ensure services received the tariff payment for patients in the same way as with individual care. In Belgium, it aimed to increase visibility of a GC trajectory in the medical record.

#### 3.4.3. Drivers

In both countries, this integration occurred after GC implementation had already started. Driven by the highly digitalised nature of scheduling and medical record systems, the absence of GC within these systems created practical challenges. Scheduling staff lacked clarity on how to book GC sessions, participants did not receive standard reminder emails as they did for individual appointments, and recruiters experienced difficulties when attempting to schedule women within the system. In the UK, an additional driver was linked to reimbursement, as care should be registered to receive the tariff payment, as illustrated below:

*“But because it wasn’t in their computer system, they weren’t being paid for it at all because, you know, there was no record that they were giving this care. So that’s one of the things that alerted us.”*

(Interview, UK)

#### 3.4.4. Impact

This modification had several impacts according to the respondents. First, it improved visibility of the GC trajectory within the medical record, supporting continuity of care across providers. Second, in the UK, it facilitated patient engagement by ensuring that participants received automated appointment reminders and avoided duplicate scheduling of individual consultations.

Establishing this integration required collaboration with, among others, the IT department, and involved several steps before it was put in practice:

*“This required significant back and forth with IT and resulted in some confusion for women and missed groups during the initial GC_1000 roll out. I was impressed by how hard the GC site steering committee worked to correct this and I think early involvement of IT for scheduling in future in a context where there is electronic scheduling is essential.”*

(Survey, UK, site 1)

As also indicated in the quote above, the need for planning in advance appeared to be underestimated. A key lesson learned was that such systems should ideally be in place prior to launching GC as a new intervention, as reflected in the following quote:

*“You have to give yourself enough time when you’re planning to make sure that’s in place. That’s, you know, you want that in place before you start recruiting. So, what we found is that really you really need to give yourself planning time.”*

(Interview, UK)

Overall, these findings indicate that early digital integration of GC into booking and medical record systems is critical to support recruitment, feasibility, visibility, and long-term sustainability within routine care pathways. Anticipating and addressing these issues prior to implementation may substantially facilitate the GC implementation process.

## 4. Discussion

Our study identified two recurring modifications across multiple countries included in this study: (1) the combination of GC sessions with individual consultations, and (2) the integration of GC sessions into the digital booking and medical record system. While the latter reflects the need to align new care models with existing administrative and digital infrastructures to ensure feasibility, the former represents a more complex modification.

The combination of GC sessions with individual consults, together with a reduced total number of GC sessions, touches on all three core components of the GC model [12]. Even though these components are integrated in the GC sessions as intended, the reduced number of GC sessions could limit opportunities to fully foster *community building*, as expressed by a UK interviewee. Participants mentioned constraints on available time, resulting in fewer topics being addressed (*interactive learning*). Furthermore, our findings suggest that it is difficult to position the GC model as a comprehensive stand-alone model of antenatal care within an obstetrician-led healthcare system (*health assessment*). In practice, rather than achieving the anticipated “best of both worlds,” this modification appeared to result in systems that could be considered as “caught between two worlds”, characterised by duplicated care, increased complexity, and reduced sustainability. The coexistence of GC and routine individual consultations may lead to overutilisation of services, increased strain on staffing and financial resources, and confusion among providers, support and administrative staff, and participants.

The observation that GC sessions were sometimes perceived as “education” whereas individual consultations were viewed as the “actual care” may reflect the social construction of clinical legitimacy in healthcare systems, where individual medical consultations are culturally and professionally positioned as the dominant and most legitimate form of antenatal care [45,46,47]. In Belgium and Kosovo, this was largely driven by the dominance of obstetrician-led care models shaping both provider and patient expectations. This resonates with sociological theories on medical dominance, which describe how physicians have historically maintained substantial control over the content and organisation of healthcare work, as well as over broader healthcare decision-making processes [45,48]. Within such systems, group-based and midwifery-led approaches may be more easily positioned as complementary rather than as comprehensive care models. These findings can also be interpreted through sociological theories on professional jurisdictions and boundary work. Professional groups continuously negotiate and defend their roles, expertise, and authority within healthcare systems [49,50]. From this perspective, the difficulties in positioning GC as a stand-alone model within obstetrician-led systems may reflect broader jurisdictional tensions between medicalised and midwifery-led models of care, including negotiations around responsibility, legitimacy, and control over antenatal care pathways [49,51,52]. Svensson argued in the late 1990s that changing healthcare contexts created greater “negotiation space” between physicians and nurses, enabling new forms of collaboration and role negotiation to emerge [52]. Our findings demonstrate that such negotiation processes remain challenging but essential to achieve implementation of complex health interventions. At the same time, the implementation process that exposed jurisdictional tensions between professional groups also appeared to create new opportunities for collaboration and mutual recognition across professional boundaries. Several sites reported improved relationships between midwives, obstetricians, and other stakeholders as implementation progressed, with increasing mutual understanding, shared decision-making, and recognition of complementary expertise. In this sense, GC implementation may function not only as a challenge to existing professional boundaries but also as a mechanism for gradually breaking them down. Such boundary-spanning collaboration has been identified as a key prerequisite for integrated care and could improve provider and patient experiences and greater coordination of services [53,54]. From this perspective, the strengthened collaboration observed in our study may itself be considered an important implementation outcome, extending beyond GC and contributing to broader efforts to restructure healthcare delivery around integrated care pathways.

This highlights the importance of establishing clear, shared care pathways and interprofessional agreements to minimise unnecessary duplication and improve clarity on care trajectories. In this regard, the Group Care site steering committee, as recommended within the GC model, may serve as a crucial platform to facilitate interprofessional collaboration, align expectations across disciplines, and support joint decision-making on how GC is embedded within existing care structures [12]. If a structured shared care pathway could be designed in collaboration with other stakeholders with clear agreements, GC may serve as a way to provide integrated care in these countries, including medical and psychosocial care, as the GC model focuses on the wellbeing of participants so they can reach their full potential [55].

The integration of GC into booking and medical record systems enhanced visibility, coordination, and feasibility of GC within routine care. At the same time, our findings underscore that such integration requires substantial planning, time, and collaboration, and cannot be expected to occur seamlessly during early implementation phases. A key lesson is that sufficient preparation time prior to roll-out is essential, as early implementation without these structures in place may lead to confusion, inefficiencies, missed appointments, and payment difficulties.

Based on the different constructs of the FRAME, we were able to derive cross-country insights regarding the goals, drivers, and impact of modifications related to scheduling when implementing GC from the perspective of our respondents. The FRAME provided insights into the modification process itself, highlighting that these modifications were often unplanned and required ongoing flexibility from implementers. Decision-making around these modifications involved multiple disciplines. A key finding was the strong influence of the overarching healthcare system, as well as the known contextual constraints such as staffing shortages and limited spaces [14,30,37]. Overall, the FRAME proved to be a valuable tool to enable a more comprehensive understanding of the range of factors that shape modifications during GC implementation processes.

In our study, we identified how the described modifications occurring in multiple countries were driven by contextual elements, and the range of implications they had. The reversal of the combined care model in the UK and the gradual strengthening of multidisciplinary collaboration observed in Belgium and Kosovo further illustrate that implementation modifications are dynamic, with their rationale, consequences, and perceived value evolving over time. These findings suggest that policymakers and practitioners should view implementation as an iterative process rather than a one-time event, incorporating mechanisms for ongoing monitoring, reflection, and adaptation. Allocating sufficient time and resources for preparatory activities and iterative modifications may be critical to support the successful integration and scale-up of complex health interventions within routine care pathways.

### Limitations and Strengths

A key strength of this study is the inclusion of semi-structured interviews alongside the survey data within the wider context of a programme that looked at context, process and experience [35,56]. These interviews provided rich context-specific insights that complemented the survey findings [56,57]. Furthermore, the inclusion of multiple countries with different healthcare contexts proved valuable to address our research questions. Nevertheless, although the inclusion of multiple countries strengthened the comparative nature of the study, the relatively small sample size and purposive sampling strategy may limit the transferability of the findings to other GC implementation contexts. Data saturation is increasingly been debated within qualitative research [58]. In line with these discussions, sample size in the present study was guided by the principle of information power rather than by an explicit aim to achieve data saturation. However, it is important to keep in mind that our results reflect site-specific situations within broader healthcare systems, but the way sites modify accordingly may differ in other non-included GC implementation sites.

Another limitation is the missing perspectives of the GC participants, i.e., the pregnant people/parents. The primary focus of this research was on the modification process of scheduling GC into regular care. As the participants are not directly involved in the implementation process and decisions, they were not included to gain insights in the modifications applied in this phase of the study. However, our findings suggest that including participants’ views on these modifications could provide valuable insights into how they perceived the modifications, as their experiences may differ fundamentally from those of the stakeholders included in the study. It is therefore important to acknowledge as a limitation that the impacts identified in this study were derived solely from our included stakeholders’ perspectives. Although these perspectives were partly informed by stakeholders’ interactions with participants, they do not represent the participants’ views directly. However, these participants’ views are included in the broader GC_1000 research study in a later phase of the study [39,40], of which this study is a part.

Another limitation relates to the assessment of model fidelity. Although the FRAME includes fidelity as one of its components, we were not able to conduct an in-depth assessment of model fidelity in this study. This was partly due to the nature of our data collection methods which were not specifically designed to capture fidelity at a detailed level. As a result, our findings provide limited insight into how the identified modifications may have affected fidelity to the GC model. This highlights a gap in existing approaches and underscores the need for more context-sensitive and methodologically robust tools to assess fidelity in complex interventions such as GC [59,60,61].

## 5. Conclusions

Our study demonstrates the value of the FRAME as a structured tool to systematically capture and interpret modifications during the implementation of GC. We were able to identify two key cross-country modifications: the combination of GC with individual consultations and the integration of GC into digital booking and medical record systems. Furthermore, our study shows similarities and differences across the implementation sites in Belgium, Kosovo and the UK in the drivers, goals, and impact of the modifications. They further underline that implementing GC is inherently a multidisciplinary process requiring ongoing flexibility and shared ownership. Collaboration among midwives, obstetricians, administrative staff, and IT services emerged as essential for sustainable scheduling within existing care pathways. To conclude, our insights emphasise the need for early planning, context-sensitive modification with model fidelity in mind, and multidisciplinary implementation efforts to support scheduling of GC in diverse healthcare systems.

## Figures and Tables

**Table 1:** Participating countries’ characteristics.

Characteristics	Belgium	Kosovo	UK
Number of participating sites	3	2	2
Type of GC	Antenatal	Antenatal	Antenatal (1) Antenatal and postnatal continuous (1)
Type of facility	Primary care (1) Hospital (1)	Hospital (2)	Hospital (1) Collaboration- antenatal care + health visitor postnatal care.
Who leads routine antenatal care	Obstetrician	Obstetrician	Midwives
GC implementation plan	six/seven antenatal GC sessions facilitated by midwives, complemented by three individual consultations with an obstetrician	eight antenatal GC sessions facilitated by midwives, supplemented with monthly individual consultations with an obstetrician	9 antenatal GC sessions organised by midwives; participants referred to an obstetrician only in cases of pregnancy complications or risks, in accordance with standard care protocols

**Table 2:** Overview of number of responses of the qualitative survey and interviews.

Country	Participating Sites	Number of Survey Responses				Interviews Completed
		Country Lead	Facilitator	Group Care Global Consultant	Total Responses	
Belgium	3	3	3	3	9	3
Kosovo	2	2	2	1	5	1
UK	2	2	1	2	5	1
**Total**	**7**	**7**	**6**	**6**	**19**	**5**

Bold: Totals number

## Data Availability

The data presented in this study are available on request from the corresponding author. To protect the participant’s privacy, the datasets with the results of the questionnaires or interview transcripts cannot be shared openly.

## Supplementary Materials

The following supporting information can be downloaded at: https://www.mdpi.com/article/10.3390/healthcare14121642/s1, File S1: the extended Framework for Reporting Adaptations and Modifications for Evidence-based Interventions (FRAME); File S2: Qualitative survey scheduling Group Care into regular care.
