# Supplementary material for: Scheduling Group Care in Routine Perinatal Care: Identifying Implementation Modifications Across Belgium, Kosovo, and the UK
Source: Healthcare (Basel). 2026 Jun 10;14(12):1642. doi: 10.3390/healthcare14121642 (PMC13299479; doi:10.3390/healthcare14121642)

Supplementary file S1 – the extended Framework for Reporting Adaptations and Modifications for Evidence-based Interventions (FRAME)

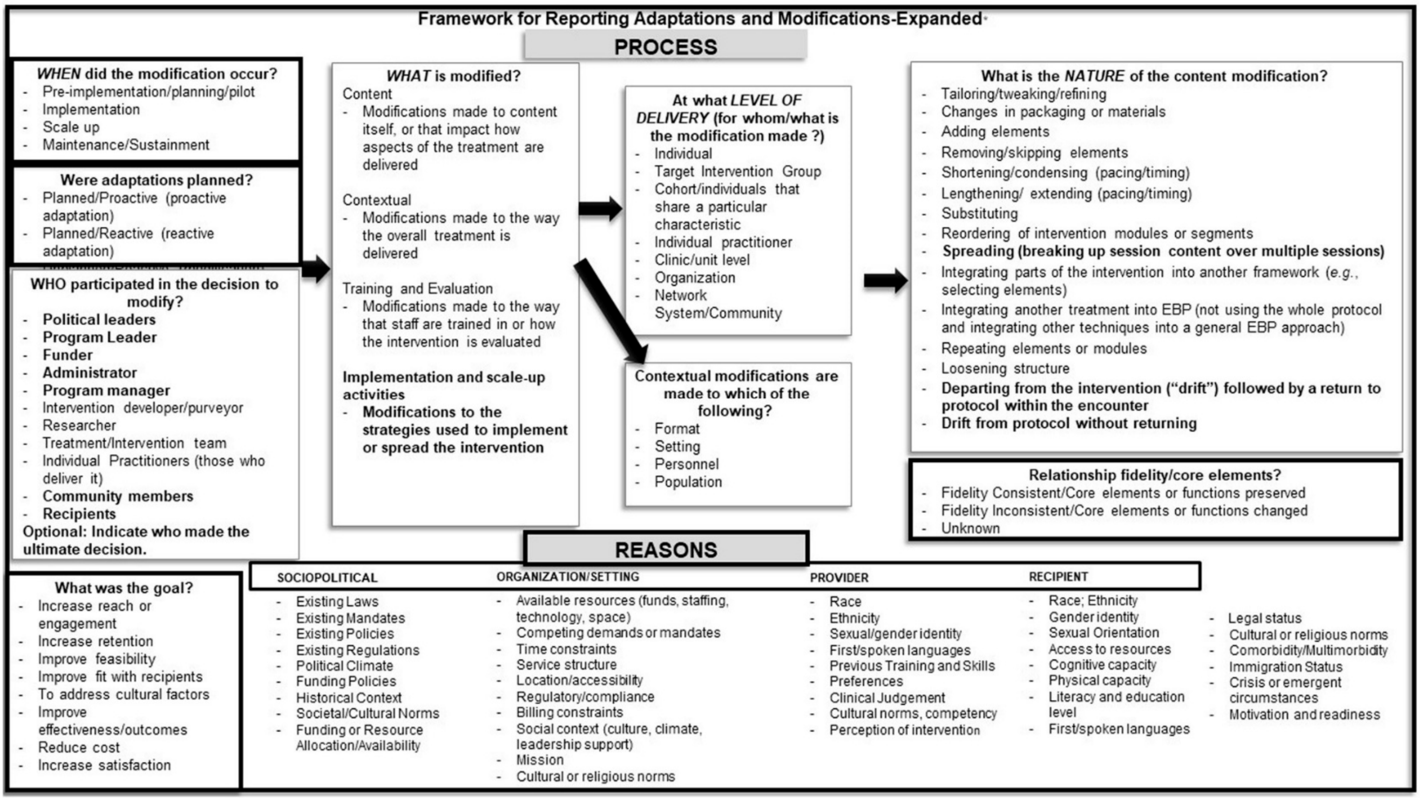

Supplement: Supplementary file 1 [file healthcare-14-01642-s001.zip › Supplementary file S1.pdf]
