# Supplementary material for: Scheduling Group Care in Routine Perinatal Care: Identifying Implementation Modifications Across Belgium, Kosovo, and the UK
Source: Healthcare (Basel). 2026 Jun 10;14(12):1642. doi: 10.3390/healthcare14121642 (PMC13299479; doi:10.3390/healthcare14121642)
Supplement: Supplementary file 1 [file healthcare-14-01642-s001.zip › Supplementary file S2.pdf]

Supplementary file S2 – Qualitative survey scheduling Group Care into regular care

Scheduling into regular antentatal/postnatal care

The second category: SCHEDULING CENTERING-BASED GROUP CARE INTO REGULAR CARE

Making the Centering-Based Group Care model **fit into the regular antenatal/postnatal care** often requires some challenging adaptations. E.g., it is not always easy or possible to adopt the 9 antenatal and 1 postnatal session as described in the Centering-Based Group Care model, and make this sessions fit with the medical check-ups indicated by protocol at the sites.

Describe **1 adaptation/action** you made in your site **regarding the scheduling of Centering-Based Group Care into regular care,** which you think of as the **most impactful** adaptation.
E.g. This can involve a change in the number of sessions that are planned in the site, or change in the organisation/logistics/... to make it possible to include the proposed number of group care sessions, or any other adaptation regarding scheduling the Centering-Based Group Care model into the regular care. If you are not aware of any adaptation in this category, please write 'I don't know'.

Thank you for describing 1 adaptation for this category "Scheduling Centering-Based Group Care into regular care". Now we move on. Please keep the adaptation you described in mind when you fill out the following questions.

SCHEDULING CENTERING-BASED GROUP CARE INTO REGULAR CARE

WHEN did the adaptation regarding scheduling Centering-Based Group Care into regular care occur?

- ◯ Before the first Centering-Based Group Care session
- ◯ Within the first year after the start of the first Centering-Based Group Care session
- ◯ After the first year after the first Centering-Based Group Care session
- ◯ I don't know
- ◯ Not applicable

Is there anything you want to add regarding WHEN the adaptation regarding scheduling Centering-Based Group Care

into regular care occured?

SCHEDULING CENTERING-BASED GROUP CARE INTO REGULAR CARE

Was the above mentioned adaptation regarding scheduling Centering-Based Group Care into regular care PLANNED?

* **Planned adaptations**: planned adaptations are typically made beforehand through a planning process that identifies ways to maximize fit and implementation success while minimizing disruption of the Centering-Based Group Care model
* **Unplanned adaptations**: unplanned reactive modifications have been defined as those that "occur during the course of Centering-Based Group Care implementation, often due to unanticipated obstacles

- ◯ Planned adaptation
- ◯ Unplanned adaptation
- ◯ I don't know
- ◯ Not applicable

Is there anything you want to add regarding if the adaptation regarding scheduling Centering-Based Group Care into regular care was PLANNED?

WHO participated in the decision to adapt regarding scheduling Centering-Based Group Care into regular care ?
Multiple answers possible.

- [ ] Group Care 1000 research team (not country-specific)
- [ ] Country research team (country lead, local researchers, ...)
- [ ] Management at the site
- [ ] Staff at the site (e.g. facilitators, logistical team at the site, IT department at site, ...)
- [ ] Pregnant women and/or their families
- [ ] Centering-Based Group Care general steering committee
- [ ] Group Care Global trainer/consultant
- [ ] Other: please specify in the extra question to add extra information
- [ ] I don't know
- [ ] Not applicable

If applicable: who made the final decision?

Is there anything you want to add regarding WHO participated in the adaptation decision regarding scheduling Centering-Based Group Care into regular care ?

SCHEDULING CENTERING-BASED GROUP CARE INTO REGULAR CARE

FOR WHOM/WHAT is the adaptation regarding scheduling Centering-Based Group Care into regular care made? *Multiple answers possible.*

- ☐ Individual - pregnant woman
- ☐ Individual - (co-)facilitator
- ☐ Target population
- ☐ Site/organisation
- ☐ Community level
- ☐ Group Care 1000 research team
- ☐ Country research team
- ☐ Health care system
- ☐ Other (please, specify)

- [ ] I don't know
- [ ] Not applicable

Is there anything you want to add regarding FOR WHOM/WHAT the adaptation regarding scheduling Centering-Based Group Care into regular care was made?

SCHEDULING CENTERING-BASED GROUP CARE INTO REGULAR CARE

What was the MOST IMPORTANT GOAL of the adaptation regarding scheduling Centering-Based Group Care into regular care?
Only one answer possible, select the best fitting option.

- ○ Increase engagement
- ○ Increase retention
- ○ Improve feasibility
- ○ Improve fit with recipients
- ○ To address cultural factors
- ○ Improve effectiveness/outcomes
- ○ Reduce costs
- ○ Increase satisfaction
- ○ I don't know

○ Not applicable

Do you consider the most important goal achieved?

○ Yes
○ Partially
○ No

Why do you consider the goal achieved/partially achieved/not achieved? Explain.

Is there anything you want to add regarding the most important GOAL of the adaptation regarding scheduling Centering-Based Group Care into regular care?

SCHEDULING CENTERING-BASED GROUP CARE INTO REGULAR CARE

Were there SOCIOPOLITICAL REASONS for adaptation regarding scheduling Centering-Based Group Care into regular care?
Select those applicable, multiple answers possible.

- ☐ Exisiting laws
- ☐ Existing mandates
- ☐ Existing policies
- ☐ Existing regulations
- ☐ Political climate
- ☐ Funding policies
- ☐ Historical context
- ☐ Societal/cultural norms
- ☐ Funding or resource allocation/availability
- ☐ I don't know
- ☐ Not applicable

Is there anything you want to add regarding SOCIOPOLITICAL REASON for the adaptation regarding scheduling Centering-Based Group Care into regular care?

SCHEDULING CENTERING-BASED GROUP CARE INTO REGULAR CARE

Were there ORGANISATION/SITE-related REASONS for adaptation regarding scheduling Centering-Based Group Care into regular care?
Select those applicable, multiple answers possible.

- ☐ Available resources, e.g. funding, staffing, technology, space, ...
- ☐ Competing demands or mandates
- ☐ Time constraints
- ☐ Service structure
- ☐ Location/accessibility
- ☐ Regulation/compliance
- ☐ Billing constraints
- ☐ Social context, e.g. culture, climate, leadership support,...
- ☐ Mission
- ☐ Cultural or religious norms
- ☐ I don't know
- ☐ Not applicable

Is there anything you want to add regarding the ORGANISATION/SITE-related REASONS for the adaptation regarding scheduling Centering-Based Group Care into regular care?

SCHEDULING CENTERING-BASED GROUP CARE INTO REGULAR CARE

Were there REASONS for adaptation regarding scheduling Centering-Based Group Care into regular care related to the (CO-)FACILITATORS of the Centering-Based Group Care sessions?
Select those applicable, multiple answers possible.

- ☐ Race/ethnicity
- ☐ Sexual/gender identity
- ☐ First/spoken languages
- ☐ Previous training and skills
- ☐ Preferences
- ☐ Clinical judgement
- ☐ Cultural norms, competency
- ☐ I don't know
- ☐ Not applicable

Is there anything you want to add regarding the REASONS for the adaptation regarding scheduling Centering-Based Group Care into regular care related to the (CO-)FACILITATOR?

SCHEDULING CENTERING-BASED GROUP CARE INTO REGULAR CARE

Were there REASONS for adaptation regarding scheduling Centering-Based Group Care into regular care related to the PARTICIPANTS of the Centering-Based Group Care sessions? *Select those applicable, multiple answers possible.*

- ☐ Race/ethnicity
- ☐ Sexual/gender identity
- ☐ Access to resources
- ☐ Cognitive capacity
- ☐ Physical capacity
- ☐ Literacy and educational level
- ☐ First/spoken language
- ☐ Legal status
- ☐ Cultural or religious norms
- ☐ Comorbidity/multimorbidity
- ☐ Immigration status
- ☐ Crisis of emergent circumstances
- ☐ Motivation and readiness
- ☐ I don't know
- ☐ Not applicable

Is there anything you want to add regarding the REASONS for the adaptation regarding scheduling Centering-Based Group Care into regular care related to the PARTICIPANTS?

SCHEDULING CENTERING-BASED GROUP CARE INTO REGULAR CARE

Relationship with the Centering-Based Group Care model

Adaptations might affect certain elements of the original Centering-Based Group Care model. Here you can read a short summary about the Centering-Based Group Care model:

Basic Model Components

- Health Care
- Interactive Learning
- Community Building

Descriptors of the Components

Health Care

The holistic health of the mother-infant dyad and family unit remains central to the group care model. Health assessment, education and promotion, and on-going evaluation are enhanced and improved through the group environment. Health

assessment is not taken from the woman or delivered to the woman as in traditional individual care, rather the health assessment happens with the woman and within the group space.

Interactive Learning

The interactive experience of the group is foundational to why groups work. Women and families are engaged in a whole new way with their care and experience during the first 1,000 days. Information is provided in the groups, and women experience and interact with the material. This interaction gives meaning and life to the topics covered in the groups.

Community Building

The consistent meeting of a cohort of women or families is also the catalyst to community building. To enhance community building, it is important that group members are consistent and that time for socializing is built into each meeting.

Did the adaptation regarding scheduling Centering-Based Group Care into regular care have an impact on the following Centering-Based Group Care aspects?

	Adaptation had a strong negative impact	Adaptation had a somewhat negative impact	Adaptation had a somewhat positive impact	Adaptation had a strong positive impact	Not applicable/ I don't know
Health assessment happens in the group space (incl. limited assessment time of 3-5 minutes)	○	○	○	○	○

	Adaptation had a strong negative impact	Adaptation had a somewhat negative impact	Adaptation had a somewhat positive impact	Adaptation had a strong positive impact	Not applicable/ I don't know
Women engage in self-care activities (e.g. measuring own blood pressure, check weight of the baby, ...)	○	○	○	○	○
There is on-going evaluation (e.g. a plan for regular assessment of outcomes)	○	○	○	○	○
Groups are facilitated to be interactive (trained facilitators / no didactic presentations,...)	○	○	○	○	○
Groups are conducted in an open circle (without a central table)	○	○	○	○	○
Each session has a plan, but emphasis may vary (E.g.Sessions are designed around the health needs of the women)	○	○	○	○	○
Groups consist of 8-12 participants, optimal for interaction	○	○	○	○	○
Group members, including facilitators and support people, are consistent (e.g. the facilitators are consistent throughout pregnancy and one is a clinician; attendance of children is discouraged)	○	○	○	○	○
There is time for socializing (e.g. unstructured time to encourages informal interaction; access to drinking water)	○	○	○	○	○

Is there anything you want to add regarding the effect of the adaptation regarding scheduling Centering-Based Group Care into regular care on the Centering-Based Group Care aspects?

The second part is finished, well done! If there is anything you want to add about the scheduling of Centering-Based Group Care into regular care, you can do it here:
